# A Two-Stage Correspondence-Free Algorithm for Partially Overlapping Point Cloud Registration

**DOI:** 10.3390/s22135023

**Published:** 2022-07-03

**Authors:** Wenhao Zhang, Yu Zhang, Jinlong Li

**Affiliations:** School of Physical Science and Technology, Southwest Jiaotong University, Chengdu 610036, China; zhangwenhao@my.swjtu.edu.cn (W.Z.); jinlong_lee@swjtu.edu.cn (J.L.)

**Keywords:** point cloud registration, overlapping regions, deep learning, global feature

## Abstract

Point cloud registration is a key task in the fields of 3D reconstruction and automatic driving. In recent years, many learning-based registration methods have been proposed and have higher precision and robustness compared to traditional methods. Correspondence-based learning methods often require that the source point cloud and the target point cloud have homogeneous density, the aim of which is to extract reliable key points. However, the sparsity, low overlap rate and random distribution of real data make it more difficult to establish accurate and stable correspondences. Global feature-based methods do not rely on the selection of key points and are highly robust to noise. However, these methods are often easily disturbed by non-overlapping regions. To solve this problem, we propose a two-stage partially overlapping point cloud registration method. Specifically, we first utilize the structural information and feature information interaction of point clouds to predict the overlapping regions, which can weaken the impact of non-overlapping regions in global features. Then, we combine PointNet and the self-attention mechanism and connect features at different levels to efficiently capture global information. The experimental results show that the proposed method has higher accuracy and robustness than similar existing methods.

## 1. Introduction

With the rapid development of laser radar and remote sensing technology, 3D point cloud data are widely used in three-dimensional reconstruction [1,2], deformation monitoring [3,4] and automatic driving [5,6]. Due to the limitation of the scanning angle of a laser scanner, the complete point cloud of an object often needs to be scanned many times. We often use point cloud registration technology that aims to predict a 3D rigid transformation to align two partially overlapping point clouds into a common coordinate system. The most commonly used registration algorithm is Iterative Closest Point (ICP) [7], which obtains the corresponding relationship through the nearest neighbor search and uses singular value decomposition (SVD) to obtain the transform matrix. However, ICP is highly sensitive to initialization and often converges to local minima. Many algorithms [8] have been proposed to solve these problems, such as Go-ICP [9], but they are usually very slow compared to ICP.

In recent years, deep learning models have made significant breakthroughs in efficiency and accuracy in many fields. Many learning-based registration methods have been proposed that can deal with large deflection angles and partially overlapping point clouds. Most of them use the deep learning method to map point clouds to high-dimensional feature space, then select the key points to be matched or construct the matching matrix through feature matching, and finally use SVD or weighted SVD to solve the rigid transformation. However, they still have some shortcomings. Deep Closest Point (DCP) [10] uses an attention-based module combining a pointer network to predict a soft matching between two point clouds, but it assumes that there is a one-to-one correspondence between the two point clouds and does not work well on partially overlapping point clouds. PRNet [11] and IDAM [12] were proposed to solve the problem of partially overlapping point clouds. However, these correspondence-based methods rely on finding accurate matching points between point clouds and easily overfit on point clouds with inhomogeneous density. The sparsity, noise and low overlap rate of real scene data make it more difficult to establish stable and accurate correspondences.

Another category of learning-based methods is global feature-based methods, such as PointNetLK [13] and PCRNet [14]. They use the knowledge of the whole point cloud by aggregating global features to register the point clouds, do not rely on the quality of key points and have high robustness to noise. These methods mainly use the feature extraction network to extract the global information of the point cloud and then use the multi-layer perceptron (MLP) to predict the rotation and translation matrix. However, such methods ignore the negative impact of non-overlapping regions and often hardly solve the problem of partially overlapping point clouds.

In this paper, we propose a novel global feature-based pipeline for partially overlapping point clouds with inhomogeneous density. In order to solve the negative impact of non-overlapping regions, we first utilize densely connected EdgeConv [15] layers to map the two point clouds to high-dimensional space; because the feature information interaction between the two point clouds is necessary, we then use a differentiable overlapping region prediction module with the attention mechanism to predict the overlapping regions. Finally, we take the sampled point clouds as the input and utilize the self-attention mechanism to capture the global information of the point clouds. We tested the algorithm on ModelNet40 [16] to verify the effectiveness of the algorithm and also tested the generalization ability of the network using the Stanford 3D Scan dataset [17], 3DMatch [18] and odometryKITTI [19].

## 2. Related Work

### 2.1. Correspondence-Based Methods

The ICP [7] algorithm solves the optimal transformation by alternating two steps iteratively: (i) estimate the correspondences between two point clouds, and (ii) estimate the rigid body transformation by solving the least-squares problem. Some variants of ICP improve performance by detecting key points [20] or weighting correspondences [21]. Nevertheless, most methods are very sensitive to initialization. Go-ICP [9] adopts a brute-force branch-and-bound scheme to search for the globally optimal solution in the pose space. Fast Global Registration (FGR) [22] uses Fast Point Feature Histogram (FPFH) [23] descriptors and an alternating optimization technique to speed up registration. However, they are either time-consuming or sensitive to noise.

Recently, many learning-based point cloud registration algorithms have been proposed. DCP [10] uses Transformer [24] to interact with the features of two point clouds and solves soft matching relationships with SVD, but it assumes that there is a one-to-one correspondence between the two point clouds. PRNet [11] extends DCP to an iterative pipeline and uses learnable Gumble-Softmax [25] to establish key-point-to-key-point correspondences. IDAM [12] uses two-stage point elimination to select key points and weight correspondences. However, the above correspondence-based methods rely on the quality and features of key points, and they are greatly affected by noise. In contrast, we aggregate the global features of the point clouds to improve the robustness.

### 2.2. Global Feature-Based Methods

PointNetLK [13] is the first method to apply deep learning to point cloud registration. It utilizes PointNet [26] to compute a global representation and optimizes the transforms by a modified Lucas and Kanade [27] algorithm. Inspired by PointNetLK, PCRNet [14] improves the robustness to noise by replacing the LK algorithm with multi-layer perceptron. However, such methods ignore the negative impact of non-overlapping regions. SCANet [28] uses channel and spatial attention to effectively use different levels of internal and global information of each point cloud and uses the features of four points to represent the features of the whole point cloud. OMNet [29] learns masks in a coarse-to-fine manner to reject non-overlapping regions; however, it is difficult to accurately estimate the masks without feature information interaction. Our network predicts overlapping regions through early information interaction to weaken the interference of non-overlapping regions.

### 2.3. Learning on Point Cloud

Recently, a large number of research papers have applied deep learning to point cloud feature descriptors, such as PointNet [26] and PointNet++ [30]. They aggregate information from individual points by using permutation-invariant pooling operations but cannot clearly represent the spatial relationship between each point. Different from point-based methods, graph-based methods do not directly use discrete points as input but construct a local region similar to a graph for each point. For example, DGCNN [31] uses dynamic graphs to capture the local geometry of point clouds. PCT [32] is based on Transformer and uses offset attention and normalization mechanisms to capture local context information in the point cloud. In this paper, we use DGCNN and Transformer to extract and interact with point cloud features, and a modified PointNet is used to extract global features.

## 3. Method

**Figure 1:**
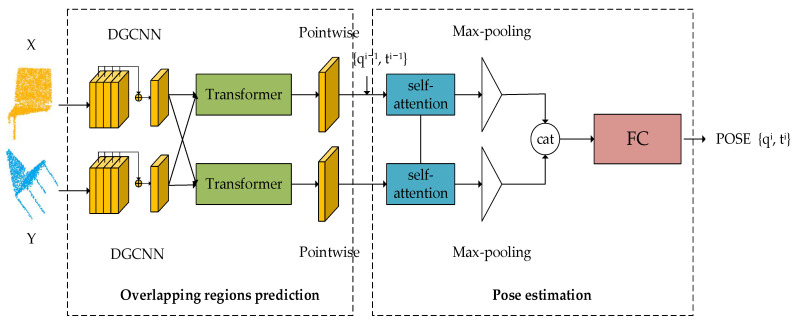
The overall pipeline of our network.

The whole framework of our network is shown in Figure 1. The network is composed of an overlapping region prediction module and a pose estimation module. Specifically, in the overlapping region prediction module, given the source point cloud $X=\left\{x_{i}\in {\mathbb{R}}^{3}|i=1,\ldots ,N\right\}$ and the target point cloud $Y=\left\{y_{j}\in {\mathbb{R}}^{3}|j=1,\ldots ,M\right\}$, we use DGCNN for feature extraction and then mix the feature information of the two point clouds to predict the possible overlapping regions. In the pose estimation module, as self-attention can efficiently capture global information [28,33], we use a feature descriptor with the self-attention mechanism to extract the global features of point clouds and bring the source cloud close to the target point cloud in an iterative way.

### 3.1. Feature Extraction

It is worth noting that we use DGCNN [31] to extract features in the overlapping region selection module, because DGCNN uses edge convolution, and the features of points contain more structural information, which makes it easier for the network to select the points in overlapping regions. However, in our pose estimation module, using edge convolution to obtain global features easily leads to over-fitting because of the inhomogeneous density of points. Therefore, we use the self-attention mechanism and operate on each point separately to obtain global features. We describe the DGCNN of point cloud X, which is the same for Y.

Firstly, we use the k-nearest neighbors (k-NN) algorithm to form graphs of the points of X in Euclidean space. Let $x_{i}^{l}$ be the feature of point $x_{i}^{}$ in the *l*th layer of EdgeConv; then, the feature of the next layer is calculated as:

(1) $$x_{i}^{l+1}=\max(\{h_{\theta }(x_{i}^{l},x_{i}^{l}-x_{j}^{l})\forall j\in N_{i}),$$

where *N_i_* represents the sum of k points closest to $x_{i}^{l}$ in point cloud X, $h_{\theta }\left(\cdot \right)$ is implemented with MLP in practice, followed by instance normalization [34] and LeakyReLU activation [35], and max(⋅) denotes element-/channel-wise max-pooling. In order to further strengthen the discriminative structure information, we connect the features of each layer. Finally, the feature $x_{i}^{f}$ of each point is expressed as follows:

(2) $$x_{i}^{f}=\max h_{\theta }(cat[x_{i}^{1},x_{i}^{2},x_{i}^{3},x_{i}^{4}]),$$

where the operation of max(⋅) and $h_{\theta }\left(\cdot \right)$ is the same as that of Equation (1), and cat[⋅] means concatenation.

### 3.2. Overlapping Region Prediction

In order to reduce the influence of non-overlapping regions on the global features of point clouds, a feasible scheme is to judge the roughly overlapping regions first. We interact with the features of the two point clouds to infer their respective overlapping regions. DCP and PREDATOR [36] have proved the effectiveness of Transformer in information interaction. In this paper, we use the attention module in Transformer to predict the overlapping regions.

Specifically, we replace the positional embedding module in Transformer with the feature information of point clouds passing through DGCNN, so the query vector *Q*, key vector *K* and value vector *V* in the attention mechanism can be defined as follows:

(3) $$Q=W^{Q}\cdot F_{X},\;K=W^{K}\cdot F_{X},\;V=W^{V}\cdot F_{X},$$

(4) $$Attention(Q,K,V)=soft\max(\frac{QK^{T}}{\sqrt{d_{k}}})V,$$

where ⋅ denotes matrix multiplication, $W^{Q}$, $W^{K}$, and $W^{V}$ are learnable weight matrices, and $F_{X}$ is the feature of point cloud X. The output attention score can be obtained from Equation (4), where *d_k_* is the dimension of key vector *K*. In practice, the number of heads in multi-head attention is 4. The features of each point cloud can eventually be updated to:

(5) $$\Phi_{X}=F_{X}^{}+transformer(F_{X}^{},F_{Y}^{}),$$

(6) $$\Phi_{Y}=F_{Y}^{}+transformer(F_{Y}^{},F_{X}^{}),$$

Then, we use the updated features to predict the probability of each point in the overlapping regions.

(7) $$S=\mathrm{soft}\max(W^{P}\cdot \Phi_{X}),$$

where $W^{P}$ is a learnable weighting matrix. We use a one-dimensional convolution layer to realize it, and the output dimension is 1.

### 3.3. Pose Estimation

PointNetLK and PCRNet both use PointNet to extract features, but PointNet operates separately for each point, which lacks the geometric knowledge of the point cloud. SCANet notes that the self-attention mechanism can effectively capture global information, so we adopted a feature aggregation method similar to SCANet.

**Figure 2:**
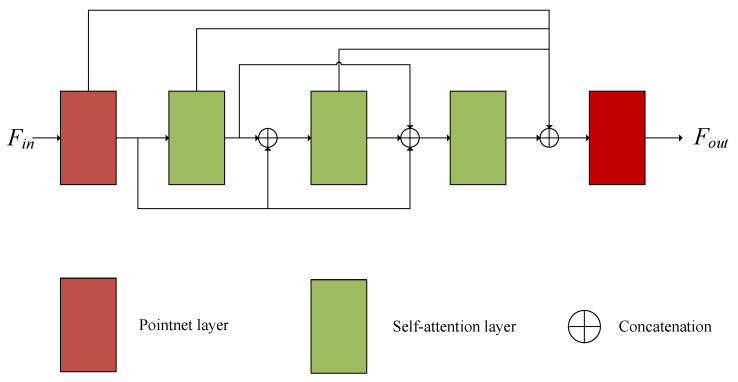
The details of global feature extraction in our method.

The framework of global feature extraction is shown in Figure 2. The input point cloud passes through five convolution modules successively. The PointNet convolution layers are used at the beginning and end, and three self-attention layers are used in the middle to enhance its structural information. Finally, max pooling is used to extract global features. In order to make use of different levels of point cloud information, we connect the features at all levels and use them as the input of the next convolution layer.

**Figure 3:**
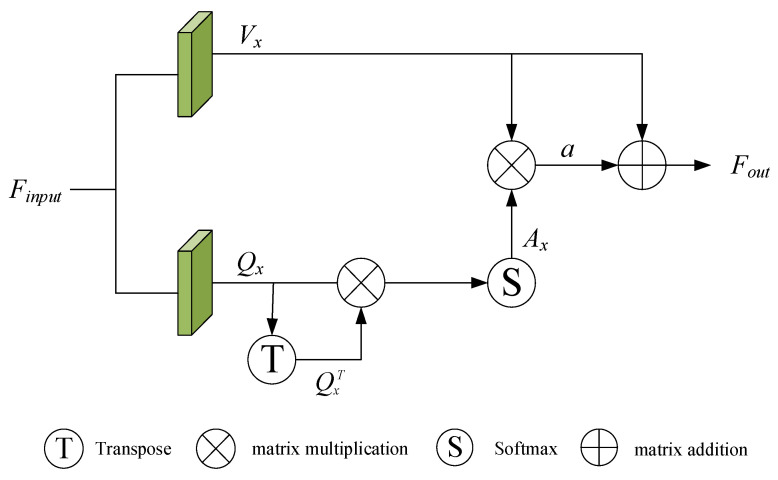
The details of self-attention block structure.

We adopted the self-attention block structure designed by SCANet. The structure diagram is shown in Figure 3. The superior input feature $F_{input}$ passes through two convolution layers to obtain a value vector $V_{x}$ and a query vector $Q_{x}$ (Equation (8)), and then the query vector $Q_{x}$ is updated to a self-attention feature $A_{x}$ (Equation (9)) through the softmax function. Finally, $V_{x}$ is combined with $A_{x}$ to obtain the final output feature $F_{out}$ (Equation (10)).

(8) $$Q_{x}={\left(W_{a}F_{input}\right)}^{T},\;V_{x}=W_{b}F_{input}$$

(9) $$A_{x}=soft\max(Q_{x}Q_{x}^{T}),$$

(10) $$F_{out}=V_{x}+\alpha V_{x}A_{x},$$

We connect the global features of two point clouds and transport them to fully connected layers with a size of (1024, 512, 256, 7). Each node of the fully connected layer is interconnected with the nodes of the previous layer, the output features of the previous layer are weighted and summed, and finally, the results are input into the activation function (Equation (11)). The final output is a 7D vector, where the first three values represent the translation vector $t\in R^{3}$, and the last four values represent the rotation quaternion $q\in R^{4}$, $q*q^{T}=1$.

(11) $$y(x)=f(\sum_{i}^{n}w_{i}x_{i}+b_{i}),$$

where $w_{i}$ is the weight coefficient in the fully connected layer, $x_{i}$ is the value of the ***i***th neuron in the previous layer, and $b_{i}$ is the offset of the fully connected layer. In practice, we use ReLU activation.

After each iteration, we take the predicted transformation as the initial pose of the input of the next iteration. After n iterations, we accumulate all previous transformations and multiply each predicted rotation transform (Equation (12)). The translation vectors are multiplied first and then added (Equation (13)).

(12) $$R_{total}^{i}=R^{i}\cdot R_{total}^{i-1},$$

(13) $$t_{total}^{i}=R^{i}\cdot t_{total}^{i-1}+t^{i},$$

where $R^{i}$ and $t^{i}$ are the rotation and translation transformations predicted in the ***i***th iteration, respectively, and $R_{total}^{i}$ and $t_{total}^{i}$ represent the overall transformation after the ***i***th iteration.

### 3.4. Loss Function

Since the performance of our network depends on the quality of the predicted overlapping regions and the accuracy of the final regression, our loss function is mainly composed of two parts.

Overlap Loss: Whether a point is located in overlapping regions is regarded as a binary classification problem, and the loss of point cloud X can be calculated as:

(14) $$L_{o}^{X}=\frac{1}{\left|X\right|}\sum_{i=1}^{\left|X\right|}{\bar{o}}_{x_{i}}\log(o_{x_{i}})+(1-{\bar{o}}_{x_{i}})\log(1-o_{x_{i}}),$$

where the ground truth label ${\bar{o}}_{x_{i}}$ is defined as:

(15) $${\bar{o}}_{x_{i}}=\left\{ \begin{array}{l} 1\;\mathrm{if}\;x_{i}\;\mathrm{in}\;\mathrm{overlap}\;\mathrm{regions}\\ 0\;\mathrm{otherwise} \end{array}\right.\;,$$

The calculation of the loss $L_{o}^{Y}$ of point cloud Y is the same. For the final overlapping loss $L_{o}$, we use the mean value:

(16) $$L_{o}^{}=\frac{L_{o}^{X}+L_{o}^{Y}}{2},$$

Transformation Regression Loss: Because quaternions are continuous, we directly supervise the predicted *q* and *t*. The transformation regression loss for iteration *i* is defined as in Equation (17), where $q_{g}$ and $t_{g}$ are quaternions of the truth rotation matrices and the truth translation.

(17) $$L_{T}=|q^{i}-q_{g}|+4\left\|t^{i}-t_{g}\right\|,$$

## 4. Experiments and Results

### 4.1. Experimental Setup

We trained our model with the Adam [37] optimizer for 250 epochs with an initial learning rate of 10^−3^. We multiplied the learning rate by 0.1 at epochs 75, 150 and 200. The network parameters were updated on a single NVIDIA GeForce GTX 1080 Ti GPU.

Following partially overlapping point cloud registration, experiments were carried out on the ModelNet40 dataset, which consists of 9843 training shapes and 2468 testing shapes from 40 object categories. In contrast to other networks, we do not use the same point cloud as the source point cloud and the target point cloud. After randomly sampling 1024 points as the source point cloud, we use the remaining 1024 points as the target point cloud and randomly generate rotations within [0°, 45°] and translations within [−0.5, 0.5], which ensure that the two point clouds are not a complete one-to-one correspondence and have inhomogeneous density. In order to generate partially overlapping point clouds, we follow the same method as [8], which randomly placed a point in space and computed its 768 nearest neighbors so that the overlap rate of generated point clouds is about 0.69.

We compared our method with traditional methods ICP [7], FGR [22] and RANSAC [38] and learning-based methods PointNetLK, DCP, PRNet, IDAM and OMNet. For ICP, FGR and RANSAC, we used the implementations in Intel Open3D [39]. For ICP, we used the default number of iterations of 30, and RANSAC has 1000 iterations. The feature extraction function used by FGR and RANSAC is FPFH. The search radius and the maximum number of neighborhood points of FPFH are 0.2 and 100, respectively. For the learning-based methods, we retrained on the same dataset. For quantitative evaluation, we used the mean absolute error (MAE (R)) and the root mean square error (RMSE (R)) for the rotation matrix. For the translation vector, we used the mean absolute error (MAE (t)) and the root mean square error (RMSE (t)).

### 4.2. Unseen Shapes

**Figure 4:**
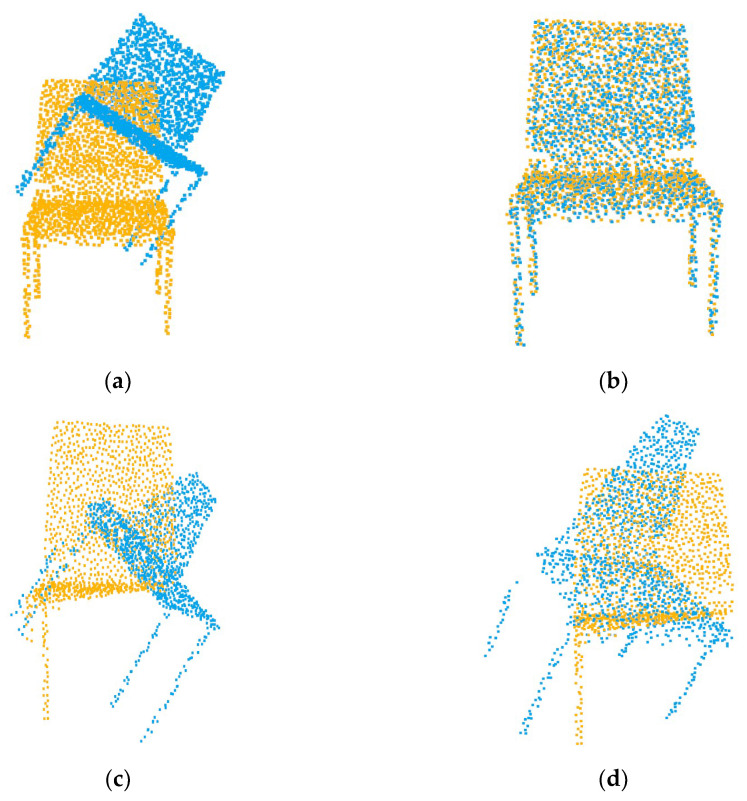
Registration results of PointNetLK with different inputs. (**a**) The fully overlapping point cloud input; (**c**) the partially overlapping point cloud input; (**b**,**d**) the respective registration results.

**Figure 5:**
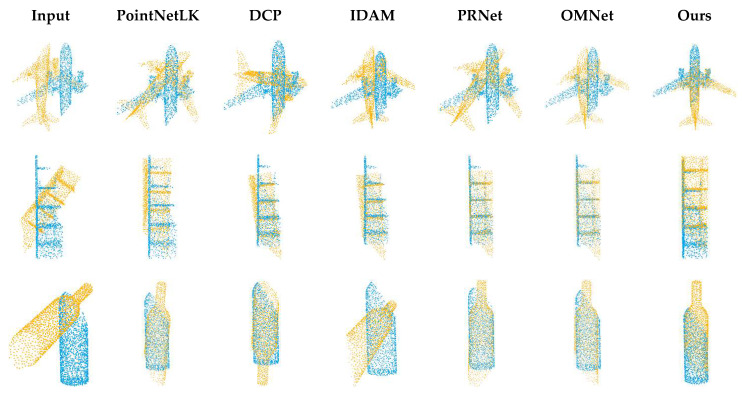
Registration visualization on ModelNet40.

Our first experiment tested the registration performance of the network at a 0.69 overlap rate. As shown in Table 1, SD(R) and SD(t) are the standard deviations of MAE(R) and MAE(t), respectively. The performance of traditional algorithms is poor due to partial overlap or a large initial pose. The registration accuracy of the global registration algorithm PointNetLK is affected by the features of non-overlapping regions. As shown in Figure 4, when we input two completely overlapping point clouds, PointNetLK performs well. However, after we input two partially overlapping point clouds, there is a large error in the registration result. The registration visualization is illustrated in Figure 5.

**Figure 6:**
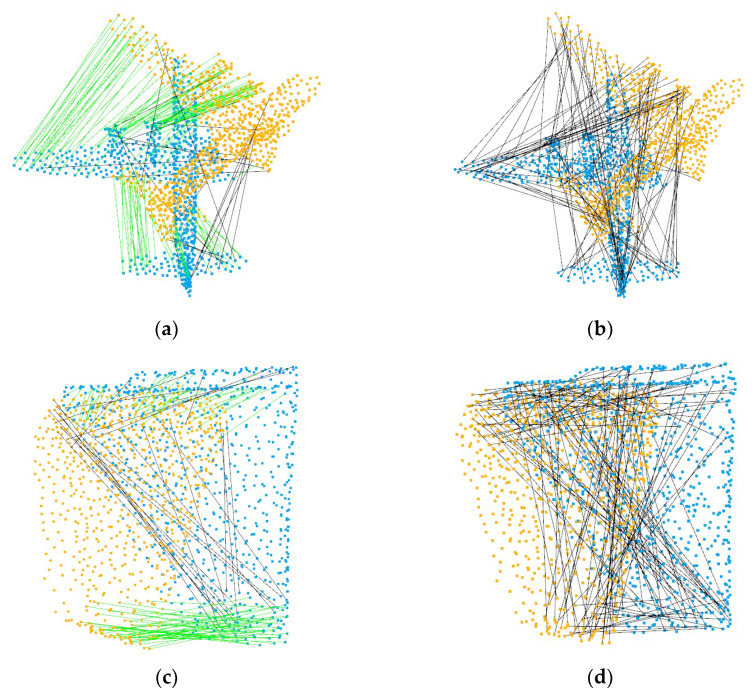
Correspondences predicted by IDAM. (**a**,**c**) Point clouds with the same distribution, (**b**,**d**) point clouds with inhomogeneous density. The green lines represent the correct correspondences, and the black lines represent the error correspondences.

For DCP, IDAM and PRNet, due to the different distribution densities of point clouds and offsets of key points, the selection of key points is not ideal, so the registration performance is not as good as that on two identical point clouds. As shown in Table 2, we just changed the method of data generation, and we obtained completely different results after retraining. As shown in Figure 6, we compare the number of correct matching correspondences selected by IDAM in the case of two kinds of data. The randomness of the data makes the network over-fit and unable to select the correct correspondences. In contrast, our method uses global features, which do not depend on the quality of key points.

### 4.3. Noise

**Figure 7:**
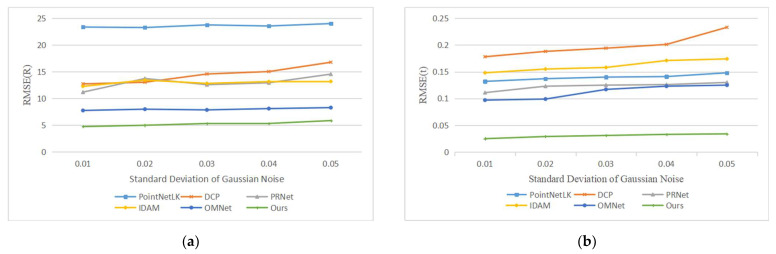
Errors of learning-based methods with different noise levels. (**a**) The experimental results of compared methods in terms of RMSE(R); (**b**) The experimental results of compared methods in terms of RMSE(t).

**Figure 8:**
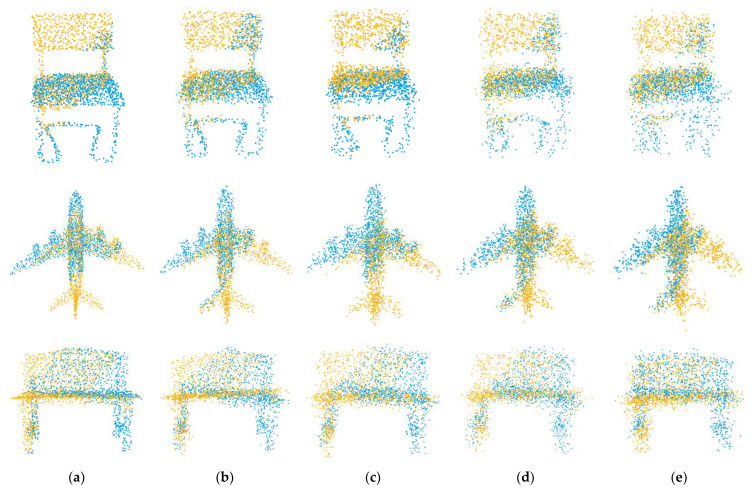
Example results on partially overlapping point clouds with varying Gaussian noise. (**a**) noise = 0.01; (**b**) noise = 0.02; (**c**) noise = 0.03; (**d**) noise = 0.04; (**e**) noise = 0.05.

Our second experiment tested the performance of the network with varying Gaussian noise. We added Gaussian noises sampled from N (0, 0.01), N (0, 0.02), N (0, 0.03), N (0, 0.04) and N (0, 0.05) and respectively clipped them to [−0.05, 0.05], [−0.1, 0.1], [−0.15, 0.15], [−0.2, 0.2] and [−0.25, 0.25] on each axis of the point clouds. It is worth noting that we used the model trained on N (0, 0.01) to test all data, rather than retraining on different noise data. The registration results are shown in Figure 7, and example results are shown in Figure 8. As the noise increases, the deformation of the two point clouds also increases, which leads to a decline in our registration accuracy, but our network still has good performance.

### 4.4. Overlap

In the following experiment, we tested the registration ability of our method on point clouds with varying overlaps. Since the number of sampling points determines the overlap rate of point clouds X and Y, we can set the number of sampling points to 768, 700, 640, 600 and 560, respectively, and the overlap rates of the generated point clouds are about 0.69, 0.58, 0.47, 0.40 and 0.32.

**Figure 9:**
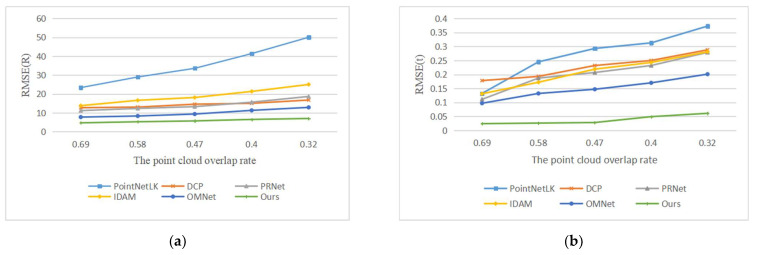
Errors of different overlapping rates. (**a**) The experimental results of compared methods in terms of RMSE(R); (**b**) The experimental results of compared methods in terms of RMSE(t).

**Figure 10:**
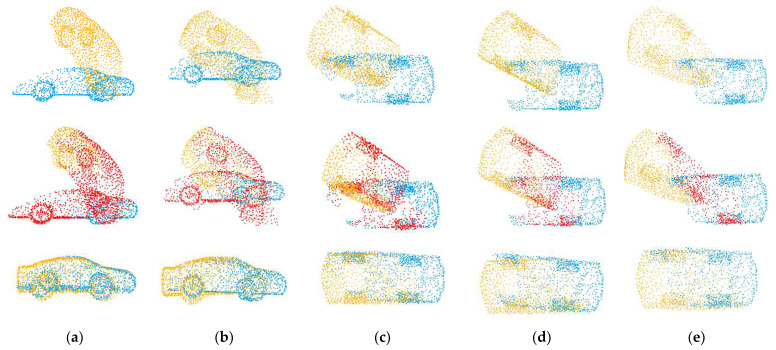
Example results on partially overlapping point clouds with varying overlap rates. The source point cloud is yellow, the target point cloud is blue, and the overlapping regions predicted by the network are red. The top row shows the initial positions of the two point clouds, and the bottom row shows the results of registration. (**a**) OR = 0.69; (**b**) OR = 0.58; (**c**) OR = 0.47; (**d**) OR = 0.40; (**e**) OR = 0.32.

We compared our method with the learning-based methods PointNetLK, DCP, IDAM, PRNet and OMNet. The registration results are shown in Figure 9, and the example results are shown in Figure 10. With the decrease in the overlap rate, the performance of all methods declines. The algorithms based on key point detection cannot effectively establish reliable correspondences due to the reduction in overlapping regions. The methods based on global features perform worse due to the interference of non-overlapping regions. Although our method has also been greatly affected, the error is far less than that of other methods. The registration results of low overlapping point clouds show that our proposed overlapping region prediction module can effectively detect the overlapping region of point clouds and greatly reduce the impact of non-overlapping regions. As shown in the second line of Figure 10, we visualize the regions selected by the network with different overlap rates.

### 4.5. Generalization

**Figure 11:**
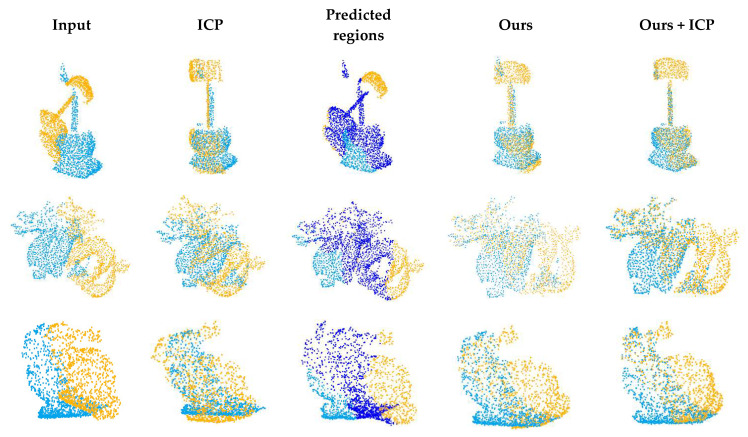
Example results on Stanford 3D Scan dataset.

Generalization is an important problem in learning-based point cloud registration algorithms. We tested the generalization of the network in this experiment. Specifically, we used the first 20 categories in the training set of ModelNet40 for training and evaluated it on the other 20 categories on the test set. The results are summarized in Table 3. We applied the experimental model to the Stanford 3D Scan dataset [17]. The results are shown in Figure 11. Since these data have not been trained, the registration accuracy is not high, but it can be adjusted as the initial position of ICP and converge to global optimization.

**Figure 12:**
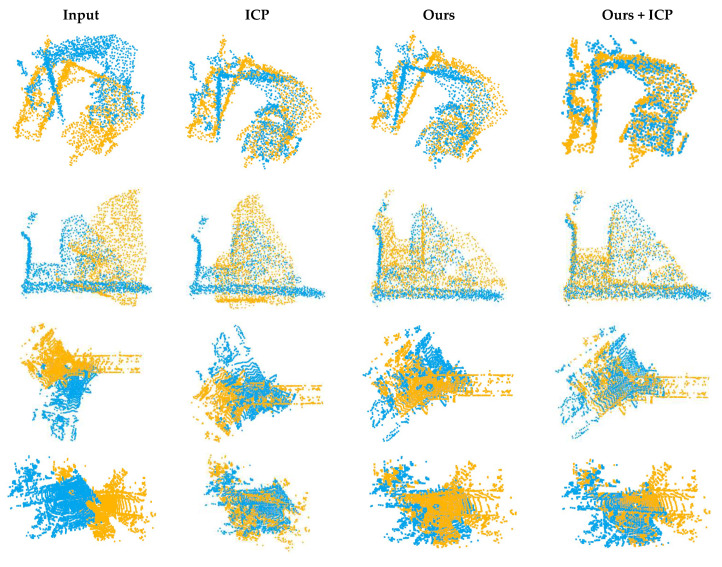
Registration results of 3DMatch and KITTI data.

In order to further test the generalization ability of the network, we applied the network to 3DMatch [18] and odometryKITTI [19]. We downsampled the original dataset to 2000–3000 points and cut the sampled data to reduce the overlapping regions of the two point clouds. As shown in Figure 12, our network can also achieve a preliminary registration effect on untrained complex scene data.

### 4.6. Ablation Experiments

In order to further analyze the performance of our network, we conducted ablation experiments. Specifically, we verified the effectiveness of each module through four sub-experiments. All experiments were carried out on the point cloud with an overlap rate of about 0.69. (1) We deleted the overlapping region prediction module and directly input the original source point cloud and target point cloud into the pose estimation module. (2) We removed the Transformer block from the overlapping region prediction module. (3) We used the combination of DGCNN and max pooling to replace our modified PointNet. (4) We replaced our modified PointNet with the original PointNet structure. We provide the resultant speed change in the network when different parts are removed. The computational time, measured in milliseconds, is obtained by averaging 100 results, and we also report the corresponding standard deviations. The results are shown in Table 4.

**Figure 13:**
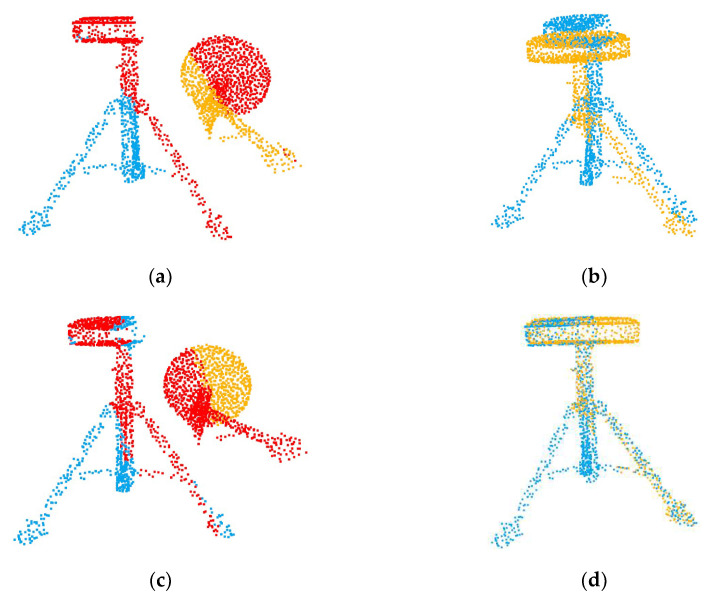
Comparison of registration results with and without Transformer. (**a**) no transformer; (**b**) registration result; (**c**) with transformer; (**d**) registration result.

The results show that if we do not have the overlapping region prediction in the first stage, our network cannot deal with the registration of partially overlapping point clouds like PointNetLK or PCRNet. Secondly, through the second comparative experiment, we can confirm that feature interaction is the key step to predict the overlapping areas of the point clouds. If there is no information interaction, the network will be able to select the overlapping regions of the source point cloud due to over-fitting, but it cannot select the overlapping regions of the target point cloud. Figure 13 shows the overlapping regions predicted by the network with or without Transformer. Through the last two sub-experiments, we also verified that our modified PointNet can better capture global features.

## 5. Discussion

In this section, we further discuss the results of the above experiments and analyze the advantages and disadvantages of our method. Previous work based on deep learning has often focused on the registration of two point clouds with the same distribution. Although they have achieved good accuracy, once applied to a point cloud with inhomogeneous density, their performance will decline sharply. Moreover, when the overlap rate is relatively low, it is difficult to select matching key points in the overlapping regions. As our experiments show, both learning-based methods and traditional key point matching methods fail to register on two partially overlapping point clouds. By gathering the knowledge of the whole point cloud, our network does not depend on the local distribution of the point cloud, so it can deal with a point cloud with inhomogeneous density and is highly robust to noise. In Section 4.3, we can see that although the two point clouds to be registered have great deformation due to noise, as long as the overall structure does not change much, our network can still achieve good performance. Secondly, unlike other global feature-based methods, we explicitly deal with the impact of non-overlapping regions. Through the overlapping region prediction module in the previous stage, we can effectively reduce the error of global features so that our network can be applied to two point clouds with a low overlap rate. Compared with similar methods, their rotational errors are more than 1.5 times that of our method, and the translation errors are more than 4 times that of our method.

However, as the overlap rate decreases, the performance of our network also decreases. We think that this is mainly affected by two factors: (1) due to the reduction in the overlap rate, the predicted overlap region has a large deviation, which seriously affects the next global registration; (2) due to the reduction in points in the overlap region, it is difficult for network training to converge to a small error. Our network also has difficulty handling two point clouds with large rotation angles. We randomly generated rotations within [0°, 180°]; however, convergence of the network becomes difficult. This is because the rotation invariance of PointNet or GNN is poor, and the features of the two point clouds can be particularly different when they are rotated by a large angle. Another disadvantage of our network is that the generalization is not good enough, which is a common problem of learning-based networks. When the network model is applied to test data that are quite different from the training data, the performance will decline. We applied the model trained on ModelNet40 to the Stanford 3D Scan dataset, 3DMatch and the outdoor KITTI dataset. The experimental results show that our network can only achieve a rough registration. Our solution is to take the predicted results of the network as the initial pose of certain fine registration algorithms, such as ICP.

## 6. Conclusions

In this paper, a two-stage network is proposed to deal with the registration of partially overlapping point clouds with inhomogeneous density. In the first stage, we combine edge convolution with Transformer to predict the overlapping regions, which aims to improve the quality of global features in the next stage. In the second stage, we combine PointNet with the self-attention mechanism to capture global information and use it to realize robust transformation regression. In contrast to previous similar work, we focus on dealing with different sources of point clouds and target point clouds with inhomogeneous density. Experimental results show that our method is highly robust to noise and can be applied to different overlap rates. We also carried out generalization experiments on the Stanford 3D Scan dataset, 3DMatch and the outdoor KITTI dataset. The experiments show that due to the over-fitting of data in the training set, the accuracy of our model will be reduced, but the results can be used as the initial pose of ICP. However, the data that we dealt with are still simple, and the number of points is small. In future work, we will combine our approach with KPConv [40] or FCGF [41] to process large scene datasets in an end-to-end manner.

## Figures and Tables

**Table 1:** Results for testing on point clouds of unseen shapes in ModelNet40.

Model	RMSE (R)	MAE (R)	RMSE (t)	MAE (t)	SD (R)	SD (t)
ICP	19.889	12.785	0.149	0.122	15.345	0.085
FGR	51.394	26.786	0.109	0.071	43.862	0.083
RANSAC + FPFH	57.596	26.278	0.087	0.043	52.016	0.072
PointNetLK	23.361	16.443	0.132	0.105	18.363	0.091
DCP	12.723	7.473	0.178	0.156	8.374	0.152
IDAM	13.789	9.447	0.131	0.097	9.149	0.093
PRNet	11.180	6.574	0.111	0.084	9.153	0.088
OMNet	7.753	5.796	0.097	0.076	4.967	0.056
Ours	4.705	2.327	0.024	0.011	3.594	0.021

**Table 2:** Results of networks with different data. Data 1 represents that the source point cloud and the target point cloud use the same data, and Data 2 represents that the source point cloud and the target point cloud have inhomogeneous density.

	Model	IDAM		PRNet	
Data		RMSE (R)	RMSE (t)	RMSE (R)	RMSE (t)
Data 1		2.839	0.019	3.372	0.020
Data 2		13.789	0.131	11.180	0.111

**Table 3:** Results of tests on point clouds of unseen categories in ModelNet40.

Category	RMSE (R)	MAE (R)	RMSE (t)	MAE (t)
First 20	4.448	2.326	0.024	0.011
Last 20	5.275	3.508	0.026	0.018

**Table 4:** Results of ablation study.

Model	RMSE (R)	MAE (R)	RMSE (t)	MAE (t)	Time (ms)	SD (Time)
No prediction	7.951	5.837	0.197	0.176	13.796	0.706
No transformer	11.688	8.579	0.184	0.156	30.304	1.392
DGCNN	14.525	11.978	0.116	0.089	42.815	1.478
PointNet	5.325	3.607	0.025	0.017	38.256	1.416
Ours	4.705	2.327	0.024	0.011	40.913	1.463

## Data Availability

The data presented in this study are openly available in [16,17,18,19].
